# Variables associated with upper extremity function in patients with Duchenne muscular dystrophy

**DOI:** 10.1007/s00415-016-8193-1

**Published:** 2016-06-17

**Authors:** Mariska M. H. P. Janssen, Jan C. M. Hendriks, Alexander C. H. Geurts, Imelda J. M. de Groot

**Affiliations:** 1 Donders Centre for Neuroscience, Department of Rehabilitation, Radboud University Medical Center, Reinier Postlaan 4, 6525 GC Nijmegen, The Netherlands; 2 Radboud Institute for Health Sciences, Department for Health Evidence, Radboud University Medical Center, Nijmegen, The Netherlands

**Keywords:** Duchenne muscular dystrophy, Upper limb, Associated variables, Regression analysis

## Abstract

Preserving upper extremity (UE) function in patients with Duchenne muscular dystrophy (DMD) is extremely important as it is related to independence and quality of life. For clinical decision making, knowledge of variables associated with UE function is necessary. This knowledge is, however, limited. Therefore, this study aims to gain more insight into the variables associated with UE function in DMD. Data from an international web-based questionnaire on UE function, obtained from 213 DMD patients, were used. Six dependent variables regarding UE function were used in multivariable linear regression analyses. In addition, 26 independent variables regarding patient characteristics, medication, therapy, supportive aids, pain, stiffness and participation were used. Twelve independent variables showed a significant relation to UE function. Variables with a negative relation to UE function were: later disease stage, occurrence of scoliosis, higher age, use of UE splints, more frequent stiffness complaints, more limitations due to stiffness, more frequent elbow pain, and having physical therapy. A positive relation with UE function was seen for going to school or work, use of corticosteroids, higher BMI, and higher age at diagnosis. These variables explained 56–81 % of the variation of the different measures of UE function. Knowledge of variables associated with UE function is very important in the clinical management of DMD patients. The results of this study suggest that corticosteroid use and participation in school and work related activities are positively related to UE function in DMD patients, as well as reducing pain and stiffness and preventing scoliosis.

## Introduction

Duchenne muscular dystrophy (DMD) is one of the most common neuromuscular disorders. DMD is an X-linked recessive disorder affecting about 1:5000 live born males [24]. The disease is characterized by progressive muscle weakening leading to functional disabilities. In an early stage boys with DMD have difficulties with walking, running and climbing stairs. Around the age of 12 they become wheelchair confined and from that age on, upper extremity (UE) function also starts to deteriorate [14, 23]. The loss of UE function leads to severe problems in the performance of daily activities and participation in society [14], ultimately affecting independence and quality of life [25].

Until now no cure has been found for DMD, however, life expectancy has rapidly increased over the last few decades. Currently, life expectancy is about 30–40 years [8, 18, 19], which means that DMD patients are in a wheelchair for the largest part of their lives and that they are fully dependent on the use of their arms during this life span. As limitations in UE function have a huge impact on the lives of DMD patients, preservation of UE function is very important. To this end, effective interventions are necessary and variables associated with UE function should be taken into consideration when making clinical decisions. Our knowledge of effective interventions and variables associated with UE function is, however, limited.

Several studies have indicated that treatment with corticosteroids has beneficial effects on the preservation of UE function in DMD patients [1, 6, 12]. In addition, Wagner et al. recommended daily stretching exercises, particularly of the distal upper extremities, in these patients [32]. However, scientific evidence for the effects of UE stretching exercises in DMD is lacking. Furthermore, evidence for the effects of physical therapy and occupational therapy on the preservation of UE function is limited. Yet, there is preliminary evidence for the efficacy of stretching and the use of splints for the lower extremities [4, 28].

To our knowledge there are no observational studies that have investigated variables associated with UE function in DMD, such as ‘participant characteristics’, ‘pain’, ‘stiffness’ and ‘participation’. However, this information could play an important role in clinical decision making with regard to the preservation (or perhaps even improvement) of UE function. Therefore, this study aimed to gain more insight into the variables associated with UE function in DMD using multivariable linear regression analysis of data obtained through a large international web-based survey [14].

## Methods

### Participant characteristics

This study was part of a larger study in which 344 participants from 14 different countries responded to a web-based questionnaire [14]. We excluded respondents that did not agree with the clinical Duchenne phenotype, based on the diagnostic criteria of Emery et al. [9]. Participants were also excluded if the diagnosis was made after the age of 10 years, or when participants who did not use corticosteroids and who were 14 years or older, were not wheelchair confined [9]. In total 213 participants were included in this study. Participants were on average 13 years (range 1–35 years) and 55 % of the participants were wheelchair confined (median age 10 years). Corticosteroid use was reported by 55 % of the respondents, while 11 % had stopped using corticosteroids and 34 % had never used steroids. In addition, 49 % of the participants had a mild or severe scoliosis. A detailed description of the participants' characteristics has been reported in a previous study [14]. This study was approved by the medical-ethical committee in the Arnhem-Nijmegen region (The Netherlands) and performed in accordance with the ethical standards laid down in the 1964 Declaration of Helsinki and its later amendments.

### The web-based questionnaire

The web-based questionnaire consisted of 224 items in total. Some items were extracted from existing questionnaires such as the capabilities of upper extremity questionnaire (CUE) [21], the ABILHAND questionnaire [31] (including few additional questions), and questions concerning pain and stiffness that were modified from the University of Michigan Upper Extremity Questionnaire [27]. Besides these existing questionnaires, questions regarding ‘patient characteristics, ‘medication’, ‘therapy’, ‘supportive aids’ and ‘participation’ were added to the web-based questionnaire.

For this study we used a subset of items from the total questionnaire (Table 1). To find the underlying dimensions and reduce the number of items for regression analysis, exploratory factor analysis was performed on the subcategories ‘pain’, ‘stiffness’, and ‘upper extremity function’ [15]. Dependent variables were the Brooke scale and the factor sum scores of the CUE and ABILHAND. Factor analysis of the CUE resulted in three factors: ‘basic hand function’, ‘heavy lifting’ and ‘light or no lifting’. ‘Basic hand function’ contains items regarding grasping and manipulating objects with the fingers. ‘Heavy lifting’ contains items regarding lifting and moving heavy objects and lifting one’s own body weight, whereas ‘light or no lifting’ contains items that require arm movements with no or minimal additional weight, such as reaching for objects or sliding light objects over a tabletop. Factor analysis of the ABILHAND resulted in two factors: ‘gross hand function’ and ‘fine hand function’. ‘Gross hand function’ contains items such as ‘washing and drying one’s hands’, ‘turning on and off a tap’, and ‘opening a lunchbox’, whereas the factor ‘fine hand function’ contains items such as ‘buttoning up a shirt’, ‘cutting nails’ and ‘inserting a key in a keyhole’. For the independent variables, factor analysis was performed on the pain and stiffness questions. Factor analysis performed on the pain questions resulted in six factors: ‘pain limitations’, ‘pain severity (not shoulder)’, ‘distal pain frequency’, ‘shoulder pain’, ‘proximal pain frequency (not shoulder)’ and ‘elbow pain frequency’. Factor analysis performed on the stiffness questions resulted in three factors: ‘stiffness frequency’, ‘stiffness limitations’ and ‘stiffness severity’. All descriptions were chosen based on the communalities of the items within one factor. Ultimately, we used the sum scores of the items within each factor for further analysis [15]. In total 32 variables were included in this study.

**Table 1:** Overview of variables

Category	Variable	Description
**Outcome measures (dependent variables)**		
Upper extremity function	Brooke	Brooke scale [2]
	Basic hand function	Sum scores of the items regarding basic hand function from the capabilities of upper extremity questionnaire (CUE) [21]^a^
	Heavy lifting	Sum scores of the items regarding heavy lifting from the CUE^a^
	Light or no lifting	Sum scores of the items regarding light or no lifting from the CUE^a^
	Gross hand function	Sum scores of the items regarding gross hand function from the Abilhand questionnaire [31]^a^
	Fine hand function	Sum scores of the items regarding fine hand function from the Abilhand questionnaire^a^
**Possible variables associated with UE function (independent variables)**		
Patient characteristics	Age	Age when participant responded to questionnaire
	Disease stage	Stage of the disease according the criteria of Bushby et al. [3]
	BMI	Body Mass Index
	Age at diagnosis	Age when the diagnosis Duchenne was established
	Injuries	Occurrence of severe injuries (e.g., bone fracture) in the arms
	Scoliosis	Occurrence of spinal deformities (e.g., scoliosis)
Medication	Corticosteroids	Use of corticosteroids
	Homeopathic remedies	Use of homeopathic remedies
Therapy	Physical therapy	Participants that receive physical therapy
	Practice at home	Participants that practice at home
	Hydro therapy	Participants that receive hydro therapy
	Occupational therapy	Participants that receive occupational therapy
Supportive aids	Splints	Use of arm/hand splints
	Arm supports	Use of arm supports
Participation	School/work	Participants that go to school or work
	Sport	Participants that participate in sports
	Hobby	Participants that practice a hobby
Pain	Pain limitations	Sum scores of the items regarding functional limitations due to pain in the arms and/or hands^a^
	Pain severity (not shoulder)	Sum scores of the items regarding pain severity in the arms and/or hands (except for the shoulder segment)^a^
	Distal pain frequency	Sum scores of the items regarding pain frequency in the wrist, fingers and thumb^a^
	Shoulder pain	Sum scores of the items regarding shoulder pain frequency and severity^a^
	Proximal pain frequency (not shoulder)	Sum scores of the items regarding pain frequency in the lower arm and upper arm^a^
	Elbow pain frequency	Sum scores of the items regarding pain frequency in the elbow^a^
Stiffness	Stiffness frequency	Sum scores of the items regarding stiffness frequency in the arms and/or hands^a^
	Stiffness limitations	Sum scores of the items regarding functional limitations due to stiffness in the arms and/or hands^a^
	Stiffness severity	Sum scores of the items regarding stiffness severity in the arms and/or hands^a^

^a^The sum scores resulted from an exploratory factor analysis that was performed on the capabilities of upper extremity questionnaire [21], the Abilhand questionnaire [31] and pain and stiffness questionnaires adapted from the University of Michigan Upper Extremity Questionnaire [27]. The complete overview of the exploratory factor analysis is described in a different study [15]

### Data analysis

Median values and ranges were used to describe the continuous variables. Valid percentages were used to describe categorical variables. Univariable regression analysis and stepwise multivariable linear regression analysis were performed to identify variables associated with the measures of UE function (dependent variables). Independent variables consisted of items from the sub categories ‘patient characteristics’, ‘medication’, ‘therapy’, ‘supportive aids’, ‘participation’, ‘pain’ and ‘stiffness’ (Table 1). Data were analyzed using IBM SPSS Statistics version 20 for Windows (IBM, Somers, NY, USA).

## Results

### Participant characteristics

In total, 213 participants were included in this study, of which 198 participants filled in the complete questionnaire and 15 participants filled in only a part of the questionnaire, as they ended the questionnaire prematurely. Table 2 describes the outcome measures that relate to UE function. Table 3 describes the possible associated variables in the subcategories: ‘patient characteristics’, ‘medication’, ‘therapy’, ‘supportive aids’, ‘participation’, ‘pain’ and ‘stiffness’.

**Table 2:** Descriptives of outcome measures

Outcome measure (min–max possible score)	*N*	Median (min–max)	Category	*N* (valid %)
Brooke	213		Brooke 1	7 (33.8)
			Brooke 2	43 (20.2)
			Brooke 3	17 (8.0)
			Brooke 4	14 (6.6)
			Brooke 5	40 (18.8)
			Brooke 6	27 (12.7)
Basic hand function (8–56)	210	48 (8–56)		
Heavy lifting (10–70)	210	31 (10–70)		
Light or no lifting (12–84)	210	57 (12–84)		
Gross hand function (15–45)	189	42 (16–48)		
Fine hand function (11–33)	191	19 (10–30)

**Table 3:** Descriptives of possible associated variables

Predictors (min–max possible score)	*N*	Median (min–max)	Category	*N* (valid %)
Age	213	13.1 (1.5–35.2)		
Disease stage	213		Early ambulatory	66 (31.0)
			Late ambulatory	29 (13.6)
			Early non ambulatory	24 (11.3)
			Late non ambulatory	94 (44.1)
BMI	209	20.1 (5.9–44.1)		
Age at diagnosis	213	4 (0–10)		
Injuries	213		No	186 (87.3)
			Yes	27 (12.7)
Scoliosis	213		No scoliosis	109 (51.2)
			Mild scoliosis	66 (31.0)
			Severe scoliosis	38 (17.8)
Corticosteroids	212		No	72 (34.0)
			Not anymore	24 (11.3)
			Yes	116 (54.7)
Homeopathic remedies	213		No	99 (46.5)
			Yes	114 (53.5)
Physical therapy	213		No	17 (8.0)
			Not anymore	19 (8.9)
			With periods of no therapy	31 (14.6)
			Yes continuously	146 (68.5)
Practice at home	213		No	123 (57.7)
			On average once a week	38 (17.8)
			On average once a day	40 (18.8)
			More than once a day	12 (5.6)
			No	92 (43.2)
Hydro therapy	213			
			Yes	121 (56.8)
Occupational therapy	213		No	123 (57.7)
			Not anymore	37 (17.4)
			With periods of no therapy	31 (14.6)
			Yes continuously	22 (10.3)
Splints	213		No	192 (90.6)
			Yes	20 (9.4)
Arm supports	213		No	195 (91.5)
			Yes	18 (8.5)
School/work	200		No	34 (17.0)
			Yes	166 (83.0)
Sport	198		No	122 (61.6)
			Yes	76 (38.4)
Hobby	198		No	34 (17.2)
			Yes	164 (82.8)
Pain limitations (0–140)	213	0 (0–140)		
Pain severity (not shoulder) (0–120)	213	0 (0–120)		
Distal pain frequency (0–36)	213	0 (0–24)		
Shoulder pain (0–32)	213	0 (0–21)		
Proximal pain frequency (not shoulder) (0–24)	213	0 (0–22)		
Elbow pain frequency (0–12)	213	0 (0–11)		
Stiffness frequency (0–84)	212	2 (0–84)		
Stiffness limitations (0–140)	212	0 (0–140)		
Stiffness severity (0–140)	212	2 (0–140)		

### Univariable regression analysis

The results of univariable linear regression analyses of potential variables associated with UE function in patients with DMD are presented in Table 4. For each dependent variable the independent variables that were associated with a *p* value <0.2 were entered in the multivariable linear regression analysis.

**Table 4:** Univariable linear regression analysis

Associated variables	Brooke		Basic hand function		Heavy lifting		Light or no lifting		Gross hand function		Fine hand function	
	*N*	*β* (95 % CI)	*N*	*β* (95 % CI)	*N*	*β* (95 % CI)	*N*	*β* (95 % CI)	*N*	*β* (95 % CI)	*N*	*β* (95 % CI)
Age	213	**0.20** (**0.18**; **0.22**)	210	**−1.34** (**−1.56**; **−1.12**)	210	**−2.06** (**−2.33**; **−1.78**)	210	**−2.86** (**−3.18**; **−2.54**)	189	**−1.23** (**−1.40**; **−1.06**)	191	**−0.69** (**−0.80**; **−0.57**)
Disease stage	213	**1.19** (**1.08**; **1.30**)	210	**−6.94** (**−8.22**; **−5.67**)	210	**−13.39** (**−14.56**; **−12.22**)	210	**−17.12** (**−18.63**; **−15.60**)	189	**−6.95** (**−7.86**; **−6.04**)	191	**−4.50** (**−5.04**; **−3.96**)
BMI	209	**0.05** (**0.00**; **0.09**)	206	0.14 (−0.22; 0.49)	206	**−0.72** (**−1.20**; **−0.23**)	206	**−0.76** (**−1.38**; **−0.13**)	185	0.05 (−0.25; 0.36)	187	−0.09 (−0.28; 0.10)
Age at diagnosis	213	**0.09** (**−0.04**; **0.22**)	210	−0.17 (−1.25; 0.90)	210	**−1.69** (**−3.14**; **−0.24**)	210	**−1.62** (**−3.50**; **0.26**)	189	0.07 (−0.87; 1.00)	191	0.08 (−0.50; 0.65)
Injuries	213	**0.79** (**0.03**; **1.54**)	210	**−5.16** (**−11.34**; **1.01**)	210	**−9.11** (**−17.51**; **−0.71**)	210	**−13.87** (**−24.60**; **−3.13**)	189	**−4.66** (**−9.78**; **0.47**)	191	**−2.33** (**−5.52**; **0.86**)
Scoliosis	213	**1.55** (**1.29**; **1.81**)	210	**−11.38** (**−13.63**; **−9.14**)	210	**−16.20** (**−19.20**; **−13.20**)	210	**−21.67** (**−25.43**; **−17.91**)	189	**−9.60** (**−11.41**; **−7.79**)	191	**−5.65** (**−6.79**; **−4.50**)
Corticosteroids	212	**−1.02** (**−1.26**; **−0.77**)	209	**6.26** (**4.16**; **8.36**)	209	**8.17** (**5.28**; **11.05**)	209	**13.83** (**10.34**; **17.32**)	188	**8.42** (**6.91**; **9.93**)	190	**5.02** (**4.07**; **5.98**)
Homeopathic remedies	213	**−0.39** (**−0.90**; **0.12**)	210	**3.38** (**−0.76**; **7.53**)	210	2.41 (−3.28; 8.09)	210	**5.90** (**−1.37**; **13.17**)	189	**2.84** (**−0.64**; **6.32**)	191	**1.42** (**−0.74**; **3.58**)
Physical therapy	213	−0.06 (−0.33; 0.21)	210	0.77 (−1.40; 2.95)	210	**−2.77** (**−5.72**; **0.18**)	210	−0.40 (−4.22; 3.42)	189	0.52 (−1.32; 2.36)	191	−0.07 (−1.22; 1.07)
Practice at home	213	0.08 (−0.19; 0.34)	210	−0.71 (−2.88; 1.46)	210	**−2.05** (**−5.00**; **0.90**)	210	−1.09 (−4.89; 2.71)	189	−0.48 (−2.29; 1.33)	191	−0.59 (−1.71; 0.52)
Hydro therapy	213	**−1.88** (**−2.32**; **−1.43**)	210	**13.23** (**9.44**; **17.01**)	210	**17.94** (**12.76**; **23.11**)	210	**27.07** (**20.72**; **33.42**)	189	**13.34** (**10.38**; **16.31**)	191	**7.33** (**5.42**; **9.24**)
Occupational therapy	213	0.03 (−0.21; 0.28)	210	0.27 (−1.73; 2.27)	210	**−1.89** (**−4.61**; **0.82**)	210	−1.51 (−5.01; 1.98)	189	−0.06 (−1.75; 1.62)	191	−0.55 (−1.58; 0.48)
Splints	213	**1.55** (**0.71**; **2.40**)	210	**−8.42** (**−15.42**; **−1.43**)	210	**−15.42** (**−24.87**; **−5.97**)	210	**−21.95** (**−34.01**; **−9.89**)	189	**−11.39** (**−16.84**; **−5.94**)	191	**−6.32** (**−9.73**; **−2.90**)
Arm supports	213	**1.76** (**0.88**; **2.64**)	210	−2.63 (−10.06; 4.79)	210	**−17.53** (**−27.40**; **−7.67**)	210	**−23.14** (**−35.78**; **−10.50**)	189	**−8.08** (**−14.08**; **−2.07**)	191	**−5.68** (**−9.39**; **−1.97**)
School/work	200	**−2.10** (**−2.74**; **−1.46**)	200	**18.63** (**13.50**; **23.75**)	200	**17.90** (**10.64**; **25.16**)	200	**29.62** (**20.56**; **38.69**)	189	**14.83** (**10.69**; **18.98**)	191	**7.73** (**5.09**; **10.37**)
Sport	198	**−0.49** (**−1.03**; **0.05**)	198	**5.76** (**1.36**; **10.17**)	198	**4.44** (**−1.50**; **10.37**)	198	**7.95** (**0.29**; **15.61**)	188	**3.59** (**0.03**; **7.16**)	190	**1.75** (**−0.46**; **3.95**)
Hobby	198	**0.90** (**0.21**; **1.59**)	198	**−4.38** (**−10.12**; **1.37**)	198	**−9.34** (**−16.93**; **−1.76**)	198	**−10.51** (**−20.39**; **−0.64**)	188	**−4.07** (**−8.77**; **0.63**)	190	−1.23 (−4.20; 1.75)
Pain limitations	213	**0.02** (**0.01**; **0.03**)	210	**−0.17** (**−0.25**; **−0.08**)	210	**−0.27** (**−0.38**; **−0.16**)	210	**−0.37** (**−0.51**; **−0.23**)	189	**−0.15** (**−0.22**; **−0.09**)	191	**−0.09** (**−0.13**; **−0.05**)
Pain severity (not shoulder)	213	**0.02** (**0.01**; **0.04**)	210	**−0.16** (**−0.27**; **−0.04**)	210	**−0.35** (**−0.50**; **−0.20**)	210	**−0.42** (**−0.61**; **−0.22**)	189	**−0.16** (**−0.25**; **−0.07**)	191	**−0.10** (**−0.16**; **−0.04**)
Distal pain frequency	213	**0.07** (**0.02**; **0.12**)	210	**−0.65** (**−1.07**; **−0.23**)	210	**−1.04** (**−1.61**; **−0.47**)	210	**−1.30** (**−2.03**; **−0.56**)	189	**−0.45** (**−0.80**; **−0.11**)	191	**−0.29** (**−0.51**; **−0.08**)
Shoulder pain	213	**0.12** (**0.08**; **0.17**)	210	**−0.92** (**−1.28**; **−0.56**)	210	**−1.50** (**−1.98**; **−1.03**)	210	**−1.87** (**−2.48**; **−1.25**)	189	**−0.86** (**−1.16**; **−0.57**)	191	**−0.48** (**−0.66**; **−0.30**)
Proximal pain frequency (not shoulder)	213	**0.09** (**0.02**; **0.16**)	210	**−0.64** (**−1.21**; **−0.06**)	210	**−1.36** (**−2.13**; **−0.59**)	210	**−1.53** (**−2.52**; **−0.53**)	189	**−0.51** (**−0.98**; **−0.04**)	191	**−0.34** (**−0.63**; **−0.05**)
Elbow pain frequency	213	**0.35** (**0.24**; **0.47**)	210	**−2.85** (**−3.78**; **−1.93**)	210	**−4.01** (**−5.26**; **−2.75**)	210	**−5.42** (**−7.02**; **−3.82**)	189	**−2.13** (**−2.89**; **−1.36**)	191	**−1.25** (**−1.73**; **−0.78**)
Stiffness frequency	212	**0.04** (**0.03**; **0.05**)	209	**−0.29** (**−0.38**; **−0.20**)	209	**−0.42** (**−0.54**; **−0.29**)	209	**−0.60** (**−0.75**; **−0.45**)	188	**−0.26** (**−0.33**; **−0.19**)	190	**−0.14** (**−0.19**; **−0.09**)
Stiffness limitations	212	**0.02** (**0.02**; **0.03**)	209	**−0.17** (**−0.22**; **−0.13**)	209	**−0.23** (**−0.30**; **−0.16**)	209	**−0.35** (**−0.43**; **−0.26**)	188	**−0.15** (**−0.19**; **−0.11**)	190	**−0.09** (**−0.11**; **−0.06**)
Stiffness severity	212	**0.02** (**0.01**; **0.03**)	209	**−0.15** (**−0.20**; **−0.09**)	209	**−0.19** (**−0.26**; **−0.11**)	209	**−0.29** (**−0.39**; **−0.20**)	188	**−0.13** (**−0.17**; **−0.08**)	190	**−0.07** (**−0.09**; **−0.04**)

Variables with a *p* value <0.2 are displayed bold. These variables were included in the multivariable regression analysis

### Multivariable regression analysis

Multivariable stepwise linear regression analysis revealed a total of 12 different variables associated with one or more aspects of UE function (Table 5). These associated variables explained 56–81 % of the variation of the different measures of UE function. The variables that were positively related to UE function were: ‘going to school or work’, ‘use of corticosteroids’, ‘higher BMI’ and ‘later age at diagnosis’. The variables that were negatively related to UE function were: ‘later disease stage’, ‘occurrence of scoliosis’, ‘higher age’, ‘use of UE splints, ‘more frequent stiffness complaints’, ‘more limitations due to stiffness’, ‘more frequent elbow pain’ and ‘having physical therapy’.

**Table 5:** Multivariable linear regression analysis

Associated variables	Brooke^a^ (*N* = 207)	Basic hand function^b^ (*N* = 199)	Heavy lifting^b^ (*N* = 199)	Light or no lifting^b^ (*N* = 208)	Gross hand function^b^ (*N* = 187)	Fine hand function^b^ (*N* = 189)
	*β* (95 % CI)	*β* (95 % CI)	*β* (95 % CI)	*β* (95 % CI)	*β* (95 % CI)	*β* (95 % CI)
Age	0.07 (0.05; 0.10)	−0.47 (−0.82; −0.12)	–	−0.99 (−1.35; −0.62)	−0.36 (−0.57; −0.15)	–
Disease stage	0.69 (0.54; 0.84)	−1.84 (−3.69; 0.02)	−10.77 (−12.19; −9.35)	−9.15 (−11.24; −7.06)	−2.69 (−3.82; −1.55)	−3.04 (−3.64; −2.44)
BMI	−0.03 (−0.05; −0.01)	–	–	–	–	–
Age_diag	−0.07 (−0.13; 0.00)	–	–	–	–	–
Scoliosis	0.31 (0.12; 0.50)	−4.79 (−7.17; −2.42)	−4.78 (−7.07; −2.49)	−4.36 (−7.11; −1.61)	−2.24 (−3.72; −0.75)	−1.30 (−2.27; −0.34)
Corticosteroids	−0.26 (−0.40; −0.12)	–	–	3.50 (1.50; 5.50)	2.76 (1.52; 3.99)	1.64 (0.82; 2.46)
Physical therapy	–	–	−2.11 (−3.66; −0.55)	–	–	–
Splints	0.45 (0.05; 0.86)	–	–	−7.04 (−12.75; −1.33)	−5.63 (−8.59; −2.68)	−2.40 (−4.43; −0.37)
School/work	–	7.34 (2.81; 11.86)	4.78 (0.61; 8.95)	–	4.04 (1.23; 6.85)	2.16 (0.44; 3.89)
Elbow pain frequency	–	−0.71 (−1.48; 0.06)	–	–	–	–
Stiffness frequency	0.01 (0.00; 0.02)	–	−0.08 (−0.16; −0.01)	−0.18 (−0.27; −0.10)	−0.09 (−0.14; −0.05)	–
Stiffness limitations	–	−0.07 (−0.11; −0.02)	–	–	–	−0.02 (−0.04; 0.00)
*R* ^2^	0.81	0.56	0.75	0.81	0.75	0.70

^a^A lower score indicates better arm function

^b^A lower score indicates worse arm function

## Discussion

The aim of our study was to gain insight into the variables associated with UE function in boys and men with DMD. Knowledge of these variables is essential for the clinical management of these patients. In this study we found four variables that were positively associated with UE function and eight variables that had a negative association with UE function.

The finding that use of corticosteroids was positively related to UE function is not surprising, as it has been proven that this medication can retard disease progression [1, 6, 12, 26]. The positive relation between going to school or work and UE function may be attributed to the fact that people that go to school or work are often physically more active than people that do not. Indeed, physical activity is important to maintain functional independence [13, 22]. The finding that patients who were diagnosed at a later age have better UE function may be due to the fact these patients usually have a slower disease progression. Another positive determinant of UE function was a higher BMI, which seems to be counterintuitive because, on the one hand, it is associated with arms that weigh more, requiring more strength to lift the arms. On the other hand, a higher BMI is often related to a better nutritional status (even though protein loss may still occur when BMI is high [16, 17]) and malnutrition occurs more often in people with a low BMI, as it is associated with dysphagia, typically occurring in the later stages of DMD [7, 30]. Malnutrition can be related to a lack of energy, increased fatigability, reduced muscle strength, and muscle wasting leading to loss of functional capacity [7, 20]. Thus, a higher BMI may be associated with a reduced likelihood of malnutrition, which could explain the positive relationship with UE function independent of disease stage. Nevertheless, future studies should try to disentangle these interrelationships to optimize clinical management.

With regard to the variables that have a negative relationship with UE function, a later disease stage and a higher age are well conceivable based on the progressive nature of DMD. Although we found no studies that related the occurrence of scoliosis to UE function, it can be expected that deformity of the spine has a negative effect on sitting balance and reduced sitting balance has a negative influence on UE function [5, 10, 11]. The negative relation of UE function with pain and stiffness is not surprising as pain and stiffness complaints are known to have a negative impact on general physical functioning [29]. However, based on our analysis, stiffness seems to have a stronger relation with UE function than pain, as only one pain variable (elbow pain frequency) was related to one dependent variable (Brooke scale), whereas stiffness variables were related to all measures of UE function. One possible explanation for the fact that stiffness seems to have a stronger relation with UE function is that DMD patients experience more stiffness-related than pain-related UE problems [14]. The fact that only elbow pain frequency relates to UE function could be because the elbow is often used as a hinge point on the arm rest or table to perform daily activities. Pain in the elbow could, therefore, be the key element in the restriction of the performance of UE activities. Remarkably, stiffness severity was not identified as a variable associated with UE function, which may indicate that stiffness severity is harder to score subjectively than stiffness frequency and stiffness limitations. Another explanation might be that the three stiffness variables were rather strongly correlated (*r* > 0.6), as a result of which stiffness severity did not add to the explained variance of UE function in the multivariable model. The finding that use of splints and physical therapy showed a negative association with UE function is probably caused by the likelihood that these interventions are recommended more often to relatively severely affected patients [4, 28]. In contrast, no relationship was found between UE function and occupational therapy, hydrotherapy or practicing at home. We hypothesize that the absence of this relation might lie in the relatively short duration of these interventions, as they are only applied for a few hours per week or even less. Therefore, exposure to therapy might not be high enough for the therapy to be effective. Going to school or work, in contrast, stimulates the use of the arm and hand over a much longer time span, which could explain its positive relation with UE function.

A limitation of this study is that our results are based on a questionnaire that was primarily designed to gain insight in UE function in patients with DMD, not for the identification of variables associated with UE function. Thus, the possible variables associated with UE function in DMD were limited to those addressed in this questionnaire, leaving the possibility that there might be other variables associated with UE function that were not investigated. Another limitation is that the cross-sectional design of our study does not allow any inferences with regard to the nature of the observed relationships (cause vs. consequence). Third, our results are entirely subjective in nature, as no objective tests of UE function, pain or stiffness were performed. Therefore, the results of this study should be interpreted with caution. Nevertheless, our study addressed 26 possible variables associated with UE function in more than 200 patients with DMD, which provides a good basis for further (longitudinal) prognostic studies, using both subjective and more objective outcome measures, to improve our understanding of the most essential variables associated with function in DMD.

It is important to realize that several of the variables associated with UE function in DMD that were identified in this study can be influenced by proper clinical management. For example, use of corticosteroids and living an active life by participating in school and work related activities can be stimulated by clinicians. In addition, prevention of scoliosis, maintaining a stable sitting balance, and reduction of pain and stiffness complaints may be attainable by regular attention from physical and occupational therapists, including the prescription of optimal assistive devices. Future longitudinal research should investigate whether proper clinical management of patients with DMD can indeed slow down the progression of UE impairments, UE activity limitations, and related participation restrictions.
